# Myosin-Va-Dependent Cell-To-Cell Transfer of RNA from Schwann Cells to Axons

**DOI:** 10.1371/journal.pone.0061905

**Published:** 2013-04-23

**Authors:** José R. Sotelo, Lucía Canclini, Alejandra Kun, José R. Sotelo-Silveira, Lei Xu, Horst Wallrabe, Aldo Calliari, Gonzalo Rosso, Karina Cal, John A. Mercer

**Affiliations:** 1 Department of Proteins and Nucleic Acids, Instituto de Investigaciones Biológicas Clemente Estable, Montevideo, Uruguay; 2 Biochemistry Section, Facultad de Ciencias, Universidad de la República, Montevideo, Uruguay; 3 Department of Genetics, Instituto de Investigaciones Biológicas Clemente Estable, Montevideo, Uruguay; 4 Cell Biology Department, Facultad de Ciencias, Universidad de la República, Montevideo, Uruguay; 5 McLaughlin Research Institute, Great Falls, Montana, United States of America; 6 Department of Biology, University of Virginia, Charlottesville, Virginia, United States of America; 7 Institute for Stem Cell Biology and Regenerative Medicine, National Centre for Biological Sciences, Bangalore, India; 8 Biophysics Area, Department of Biochemistry, Molecular and Cell Biology, Facultad de Veterinaria, Universidad de la República, Montevideo, Uruguay; Hertie Institute for Clinical Brain Research, University of Tuebingen, Germany

## Abstract

To better understand the role of protein synthesis in axons, we have identified the source of a portion of axonal RNA. We show that proximal segments of transected sciatic nerves accumulate newly-synthesized RNA in axons. This RNA is synthesized in Schwann cells because the RNA was labeled in the complete absence of neuronal cell bodies both *in vitro* and *in vivo*. We also demonstrate that the transfer is prevented by disruption of actin and that it fails to occur in the absence of myosin-Va. Our results demonstrate cell-to-cell transfer of RNA and identify part of the mechanism required for transfer. The induction of cell-to-cell RNA transfer by injury suggests that interventions following injury or degeneration, particularly gene therapy, may be accomplished by applying them to nearby glial cells (or implanted stem cells) at the site of injury to promote regeneration.

## Introduction

The existence and extent of axonal protein synthesis has been a contentious issue for decades, but evidence supporting it has steadily accumulated. In turn, this raises the question of whether the mRNAs translated in the axon are transcribed in the cell body, glia, or both [1]–[7]. In recent years, evidence from multiple sources supports the hypothesis that Schwann cells in the peripheral nervous system transfer messenger RNA and ribosomes to the axons that they ensheath. Early evidence suggested transfer of newly-synthesized RNA and/or protein from Schwann cells to axons [8]–[12]. Later studies showed the presence of neurofilament subunits and the mRNAs that encode them in Schwann cells [13]–[17], suggesting mRNA transfer to axons because these proteins are considered to be axonal-specific proteins. Morphological evidence also has suggested cell-to-cell transfer of ribosomes [18]–[20]. The most conclusive evidence for ribosomal transfer comes from expression of a tagged ribosomal protein in sciatic nerves of *Wld^s^* mice following injury [21], and in regenerating nerves of normal mice [22].

**Figure 1:**
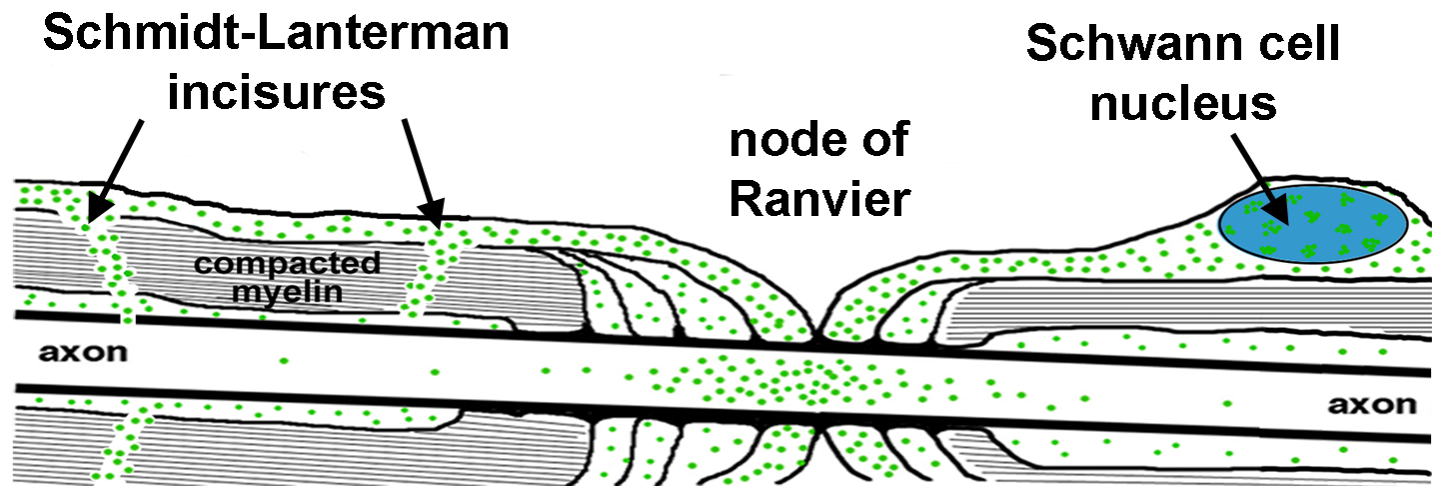
Possible routes for transfer of newly-synthesized RNA from Schwann cells to axons. Diagram of a peripheral fiber showing a longitudinal section of parts of two adjacent Schwann cells and the axon they ensheath. This schematic depicts hypothesized routes (nodes of Ranvier and Schmidt-Lanterman incisures) of transport of BrU-labeled RNA (green dots) between the Schwann cell nucleus and the axon.

The present study shows that axons proximal to transections of rat and mouse sciatic nerves accumulate newly-synthesized RNA that is labeled by bromouridine in the absence of the neuronal cell bodies. The shortest, quickest routes for such RNA to travel from the Schwann cell nucleus to the axon are via the nodes of Ranvier or Schmidt-Lanterman incisures (Fig. 1), also suggested by Twiss and Fainzilber [6]. This BrU-labeled RNA is tightly packaged and F-actin is required for its transfer to axons. We also show that myosin-Va function is required for transfer, as homozygous *Myo5a* null mutant mice fail to accumulate newly-synthesized RNA in axons. Our results conclusively demonstrate cell-to-cell transfer of RNA. They also suggest that the mechanism of transfer may be similar to the mechanism by which melanosomes are transferred from melanocytes to keratinocytes, which also is disrupted to produce the diluted coat color of myosin-Va-deficient mice.

## Materials and Methods

### Ethics Statement

All mouse work performed at the McLaughlin Research Institute (MRI) was carried out in strict accordance with the recommendations in the Guide for the Care and Use of Laboratory Animals of the National Institutes of Health. The protocol was approved by the Institutional Animal Care and Use Committee (Protocol JAM-32). All surgery was performed under isoflurane anesthesia and all efforts were made to minimize suffering. MRI is fully accredited by AAALAC. All rat and mouse work performed at the Instituto de Investigaciones Biológicas Clemente Estable (IIBCE) was carried out in strict accordance with that institution's Comité de Ética en el Uso de Animales (CEUA-IIBCE) under law 18.611 of the República Oriental del Uruguay. The specific protocol was approved by the CEUA-IIBCE (Protocol Sotelo-013/09/2011). All surgery was performed under pentobarbital anesthesia and all efforts were made to minimize suffering.

### Sciatic Nerve Transection

**Figure 2:**
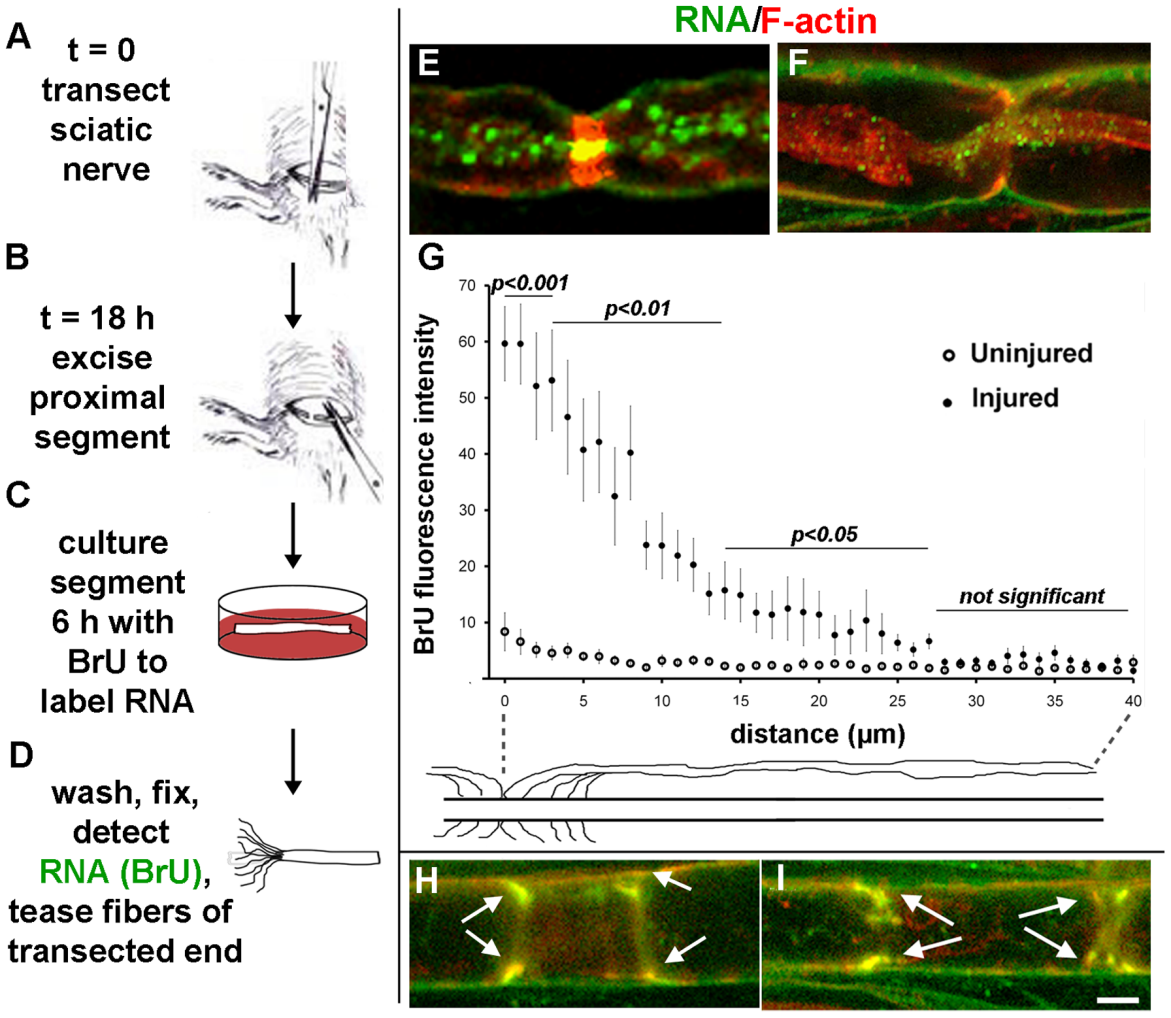
Newly-synthesized RNA is transferred from Schwann cells to axons after sciatic nerve transection. **A–D**, experimental procedure. **E–F**, single confocal planes of fibers at nodes of Ranvier showing BrU incorporation (green) and F-actin (red). **G**, Axonal BrU fluorescence intensity plotted as a function of distance from the node of Ranvier for uninjured control (open circles) and injured (closed circles) nerves. Statistical significance at each distance between injured and uninjured nerves was determined by Student's t-test. Error bars represent standard errors. **H–I**, single confocal planes showing BrU labeling (green) of F-actin-rich (red) Schmidt-Lanterman incisures (arrows). Bars = 5 µm.

Adult Sprague-Dawley or Wistar rats were anesthetized with 50 mg/kg pentobarbital. An incision was made at mid-thigh and the sciatic nerve was transected (diagram, Fig. 2A). Incisions were closed with cyanoacrylate glue. After 18 h recovery, the rats were euthanized and a 2-cm sciatic nerve segment proximal to the transection was removed (Fig. 2B); equivalent contralateral uninjured segments were used as negative controls. The segments were incubated in Neurobasal medium (Invitrogen) containing 2.5 mM bromouridine (BrU, Sigma) for 1, 3 or 6 h at 37°C, 5% CO_2_ (Fig. 2C). Representative nodes of Ranvier for all three time points are shown in Fig. S1 in File S1. Only 6-h incubations are shown in all other figures. A negative control in which transected nerve segments were incubated for 6 h in Neurobasal medium lacking BrU also was performed. As an *in situ* control for artifacts that might be caused by explanting the nerve segments for BrU labeling, transection of both sciatic nerves was followed by a proximal crush injury (achieving axonotmesis) after 18 h, instead of the second transection and explantation shown in Fig. 2. BrU was then applied in situ to the left sciatic nerve in the thigh for 3 h under anesthesia [10]. Meanwhile, the injured contralateral nerve was explanted and incubated in BrU for 3 h. In all experiments, segments were washed 10 times for 5 min each in ice-cold PHEM buffer (60 mM PIPES, 25 mM HEPES, 10 mM EGTA, 2 mM MgCl_2_) to remove unincorporated BrU, then fixed for 30 min in 3% paraformaldehyde in PHEM at room temperature. Segments were treated for 1 h at 37°C with 0.2 mg/ml collagenase (Sigma) in PHEM with 5 mM CaCl_2_ and without EGTA. The nerve fibers were released from epineurium with #5 forceps and teased at the injured end with 26-gauge needles (Fig. 2D). The segments were permeabilized with 0.1% triton X-100 in PHEM buffer for 30 min at room temperature.

### Immunocytochemistry

The incubation buffer for all steps was 0.1% BSA and 50 mM glycine in PHEM buffer. Nerve segments were prepared for immunocytochemistry by blocking in 5% normal goat serum for 30 min at 37°C. Permeabilized fibers were incubated with anti-BrdU (Sigma, 1∶300), anti-CASPR (Abcam, 1∶300), anti-myosin Va (kindly supplied by Roy Larson, 1∶100), or antiserum against purified ribosomes [20] (1∶1000) for 1 h at 37°C. Fibers were washed 6 times 5 min each. Secondary antibodies (goat anti-mouse or goat anti-rabbit conjugated with Alexa 488, 546, or 633, all from Invitrogen, all 1∶1000) were incubated for 45 min at 37°C. F-actin was detected using fluorescent phalloidin (Invitrogen) added together with secondary antibodies. Fibers were then washed 6 times 5 min each. Finally, individual fibers were teased and mounted in ProLong Antifade (Invitrogen).

### α-Amanitin Treatment

RNA polymerase II was inhibited by adding 10 µg/ml α-amanitin (Sigma) during the BrU labeling step described above.

### Ribonuclease Treatment

After the wash step to remove soluble BrU, sciatic nerve segments were incubated with RNAse in PHEM buffer at 5 or 10 mg/ml for 1 h, at 37°C. Segments were washed 10 times 5 min in PHEM at room temperature.

### Latrunculin A Treatment

F-actin was depolymerized by the addition of 0.07, 0.2, 0.6, or 1.8 µg/ml Latrunculin A (Sigma) during the BrU labeling step.

### *In situ* Hybridization

Incubations were performed at room temperature unless otherwise stated. Frozen 10-µm sections of uninjured mouse sciatic nerves were blocked with 0.03% H_2_O_2_ for 1 h, washed 3 times 5 min in 4X SSC, and prehybridized in 4X SSC, 50% formamide, 10% dextran sulfate, 0.1 mg/ml tRNA, and 0.5 mg/ml sheared salmon sperm DNA for 2 h at 54°C. Hybridization was carried out for four hours at 54°C in the same buffer plus 0.5 ng/ml of *in vitro* transcribed digoxigenin labeled probe complementary to the small subunit of neurofilament mRNA (nucleotides 1858 to 1959, NM_010910). Sections were washed twice for 10 min in 4X SSC plus 30% formamide at 54°C, then twice for 5 min each in 2X, 1X, 0.5X, and 0.25X SSC. Sections were postfixed in 3% paraformaldehyde in PHEM for 5 min and washed three times for 5 min in PHEM. Blocking was performed as described for immunocytochemistry above. Incubation with primary antibodies (mouse anti-BrdU, HRP-Sheep anti-digoxigenin) was performed overnight at 4°C. Sections were washed 3 times for 10 min in PHEM and then incubated in tyramide amplification reagent according to the instructions of the manufacturer (Invitrogen) for 10 min. Excess tyramide was removed by washing 3 times for 5 min with PHEM. Secondary antibody (Goat anti-mouse Alexa 546 and goat anti-rabbit Alexa 633, Invitrogen) incubations were performed for two hours. Three washes for 5 min with PHEM were performed before mounting in ProLong (Invitrogen).

### Confocal Microscopy

Teased fibers were visualized with an Olympus FV-300 confocal microscope, equipped with a Plan Apo N 60X oil NA 1.42 lens and 488, 543 and 633 nm laser lines. Images were processed with Fluoview and ImageJ software. Nodes of Ranvier chosen for quantitative analysis were all within 100 µm of the injured end.

### FRET Analysis

To estimate the distance between myosin-Va and newly-synthesized RNA, we performed quantitative fluorescence resonance energy transfer (FRET) between the secondary antibodies recognizing the primary antibodies described above. Images were collected for FRET analysis using single-labeled donor or acceptor samples and double-labeled samples: 4 single-label donor reference images (donor excitation in both donor and acceptor channels); 4 single-label acceptor reference images (donor and acceptor excitation, both in the acceptor channel); six double-label images (donor excitation in donor and acceptor channels, acceptor excitation in acceptor channel). FRET analysis was performed using the precision FRET (PFRET) algorithm plugin for ImageJ [23]–[25]. Additional images of nonlabeled samples were taken for background subtraction of autofluorescence. Twenty nodes of Ranvier were analyzed in two separate experiments. The selection of appropriate ROIs was made automatically by ImageJ software. Supporting data are shown in Fig. S3 in File S1.

### Mouse Sciatic Nerve Transections

The mouse experiments were performed as described above for rats, with the following differences: Age-matched 12–16-day-old C57BL/6J control and *Myo5a^d-l20J^/Myo5a^d-l20J^ (dilute-lethal)* null mutant mice were anesthetized with isoflurane in oxygen before transection. After euthanasia on the following day, a ∼3-mm proximal segment was removed and cultured in BrU. After immunocytochemistry, segments were frozen in OCT and 10-µm longitudinal sections were mounted on slides for confocal microscopy using an Olympus FV-1000. Intraperitoneal injection of the mice with BrU gave identical results to *in vitro* culture, controlling for artifacts that might be caused by the *in vitro* BrU labeling protocol. Intraperitoneal injection also was performed on older (2 mo) wild-type mice and teased fibers were examined as described for rats above.

## Results

### RNA Transfer from Schwann Cells to Axons

To assay for cell-to-cell transfer of RNA, newly-synthesized RNA was labeled with BrU in a rat sciatic nerve transection protocol (Figure 2A–D). This protocol separates the axons from their cell bodies, making it impossible for the neuronal nucleus to be the source of any newly-synthesized RNA imaged with BrU. Within the nerve segments, we observed two general classes of fibers: those that had little or no BrU signal, likely representing dead or dying fibers that did not survive the injury and explantation, while the other class had robust BrU signals (green).

The most prominent labeling observed was a punctate labeling of axons at nodes of Ranvier (Figure 2E and F). This label gradually decreased with distance from the node (Fig. 2G). The gradient of BrU signal from the nodes of Ranvier to 40 microns in each direction is plotted in Fig. 2G. Analyzing injured vs. uninjured axons at each distance by Student’s t-test, the values were statistically different between 0–2 µm (*p<*0.001), 3–13 µm (*p<*0.01), and 14–27 µm (*p<*0.05). Another possible path for transport of material between Schwann cells and axons is through Schmidt-Lanterman incisures [6]. We saw extensive BrU labeling of these as well (Fig. 2H and I, arrows).

**Figure 3:**
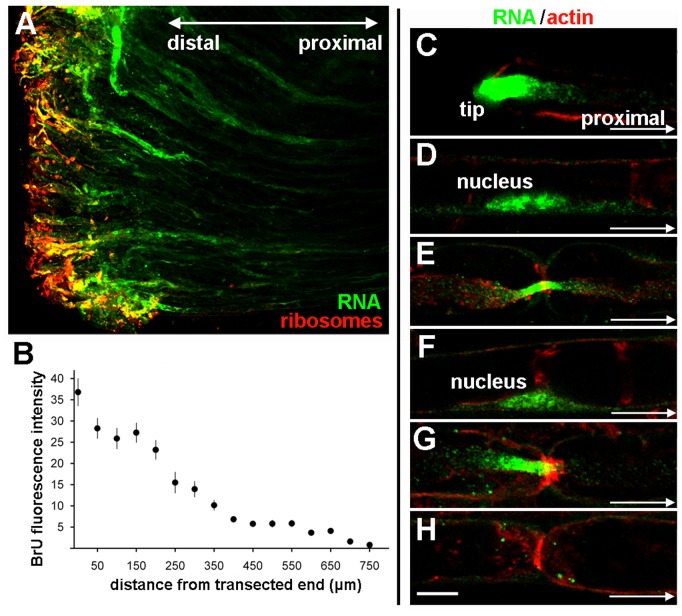
Levels of newly-synthesized RNA decline as a function of distance from nerve injury. **A**, low-magnification micrograph of transected end showing newly-synthesized RNA (green) and ribosomes detected by anti-P antibody (red). Bar = 100 µm. **B**, BrU-RNA signal plotted as a function of distance from the transection. Each point represents the mean of 10 nerve fragments with standard errors. **C–H**, series of images of a single fiber from the transected end, distal to proximal, showing newly-synthesized RNA labeled by BrU (green) and F-actin (red). **C**, transected end with a high concentration of newly-synthesized BrU-RNA. **D**, first proximal Schwann-cell nucleus from the tip. **E**, first node of Ranvier proximal from the tip. **F**, second Schwann cell nucleus. **G**, second node of Ranvier. **H**, third node of Ranvier. Bar = 10 µm.

At lower magnification, the heterogeneity of BrU labeling in individual fibers at the injured end of the nerve segment and the distal-proximal gradient of labeling from the transection site is shown in Fig. 3A (green). The concentration of ribosomes is greatly increased (red), but the ribosomal distribution is only partially coincident with the newly-synthesized RNA distribution. The BrU gradient over the 750 µm from the transection site is quantified in Fig. 3B. A distal-to-proximal series of a single representative labeled fiber is shown in Fig. 3C–H, counterstained with fluorescent phalloidin to label F-actin (red). In this single fiber, we observe BrU labeling prominent in axons at the injured tip (Fig. 3C), punctate labeling at nodes of Ranvier (Fig. 3E and G) and in the nuclei (Fig. 3D and F) and outer cytoplasmic wraps of the Schwann cells. The most proximal micrograph (Fig. 3H) shows that the nodal labeling tends to decrease as a function of distance from the injured end, as it lacks axonal RNA.

**Figure 4:**
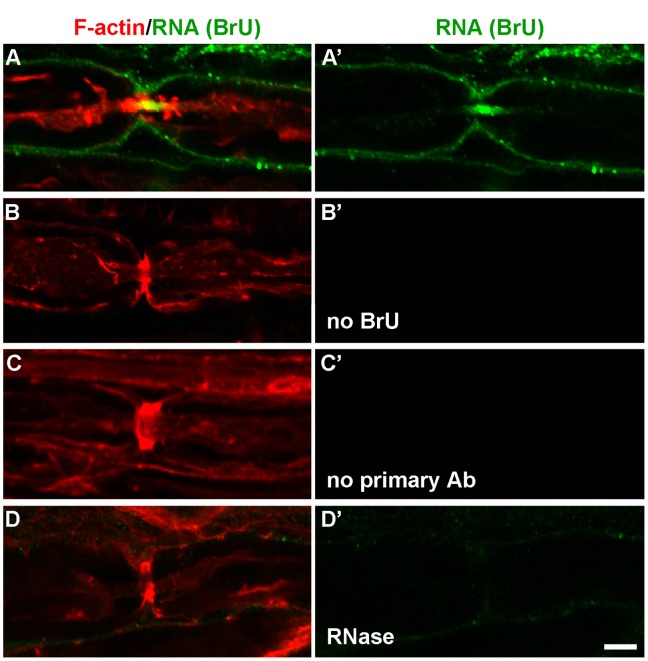
Negative controls for observation of axonal BrU labeling. In all panels, newly-synthesized RNA is shown in green, with F-actin counterstaining shown in red. **A and A’**, experimental condition; fibers were incubated with BrU. **B and B’**, negative control incubated with medium without BrU. **C and C’**, negative control incubated in BrU, but primary anti-BrdU antibody was omitted. **D and D’**, negative control incubated with 10 mg/ml RNAse. Both BrU and F-actin channels at a single confocal plane are shown in **A**–**D**, whereas the BrU channel alone is shown in **A’**–**D’**.

**Figure 5:**
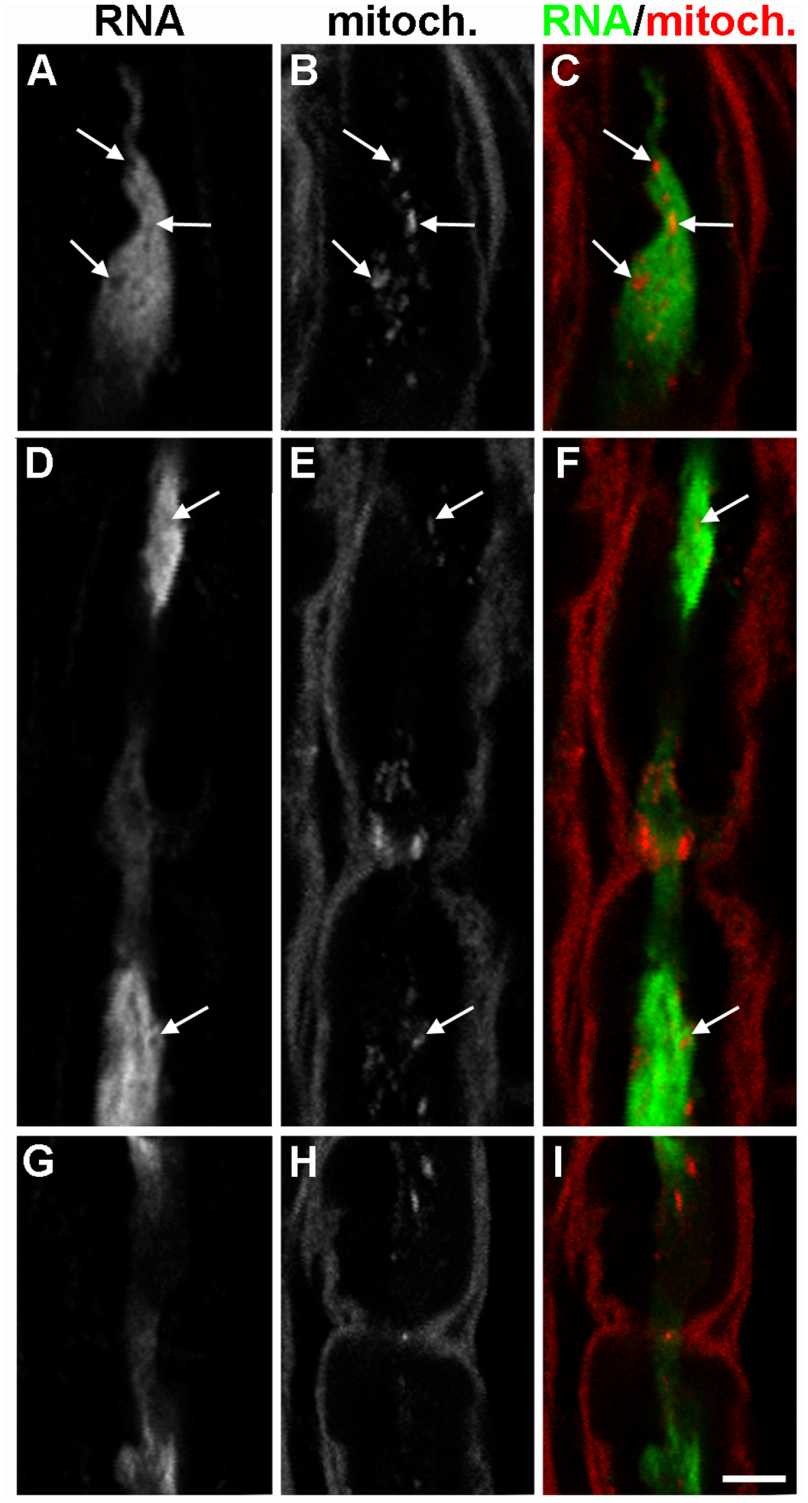
Most newly-synthesized axonal RNA is not mitochondrial. Cryosections of injured BrU-labeled (green) sciatic nerve fragments were stained for BrU **(A, D, G)** and a monoclonal antibody against the mitochondrial Complex IV Subunit I **(B, E, H)**. A paranodal axon is shown in **A–C** and nodes of Ranvier are shown in **D–I**. Mitochondria corresponding to empty spaces in A and D are designated by arrows. Bar = 5 µm.

To ensure that the immunoreactivity we detected was actually due to the incorporation of BrU into RNA synthesized in Schwann cells, we performed a series of negative controls (Fig. 4) in addition to the uninjured negative control (Fig. 2G). The standard conditions are shown in Fig. 4A. The negative controls included performing the procedure in the absence of BrU (Fig. 4B), without primary anti-BrU antibody (Fig. 4C), and with 10 mg/ml ribonuclease A (RNase A) (Fig. 4D). All showed little or no BrU labeling of Schwann cells or axons. Consistent with packaging of the labeled RNA, 5 mg/ml RNase only reduced the BrU signal (data not shown), but 10 mg/ml eliminated it altogether. We also performed the procedure without allowing any time for incubation in BrU to control for nonspecific binding/aggregation of BrU (no labeled RNA was detected, data not shown). To eliminate the possibility that the axonal BrU labeling we observed originated in axonal mitochondria, we labeled mitochondria with antibody raised against complex IV subunit 1 (Fig. 5). There was little overlap between the mitochondrial marker and the BrU signal, indicating that the majority of RNA we observed was not of mitochondrial origin. More importantly, mitochondria appeared as “holes” in regions with high BrU signal (arrows in Fig. 5), suggesting no colocalization. Finally, to show that the observation of labeled axonal RNA was not an artifact of the explant protocol, we performed the labeling in the rat thigh after transection and a crush injury 18 h later, followed by 3 h labeling *in vivo* and in situ (Fig. S2 in File S1). The gradient of BrU labeling from the transection site and the distribution at the nodes of Ranvier were indistinguishable from those observed with *in vitro* labeling. Together, these controls conclusively demonstrate that we are observing transfer of newly-synthesized RNA from Schwann cells to axons.

**Figure 6:**
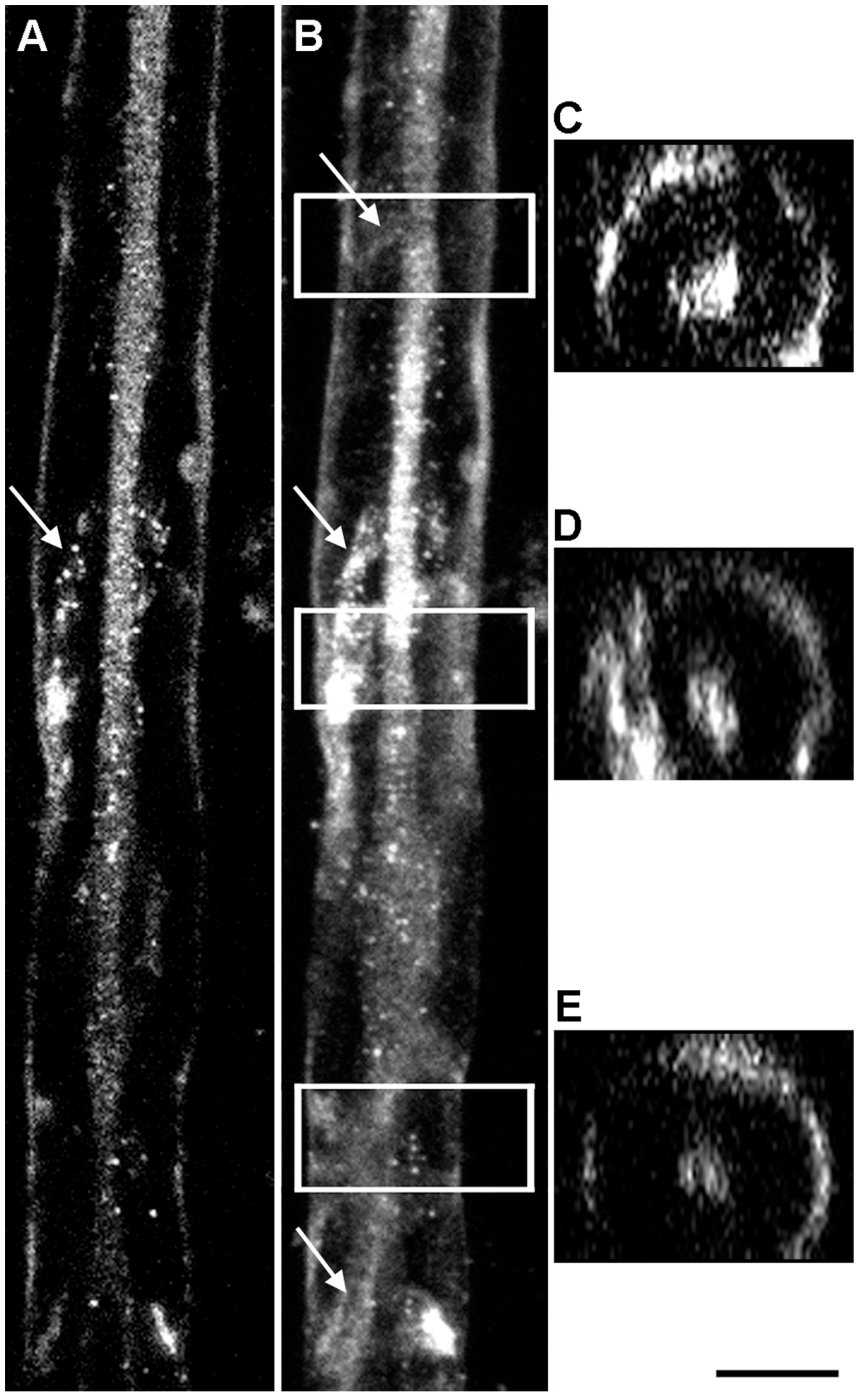
Newly synthesized RNA is present in axons and bands of Cajal. **A**, confocal plane including a BrU-labeled axon. The myelin is unlabeled. The external border of the myelin is the outer wrap of Schwann cell cytoplasm that includes bands of Cajal. **B**, stack of confocal planes with the plane shown in A as the midpoint, showing the spiraling bands of Cajal (arrows). **C, D, E**, projected cross-sections boxed in the stack shown in panel B showing the separation between newly-synthesized RNA in the axon and band of Cajal. Bar = 10 µm.

To demonstrate spatially that the axons are labeled with BrU, we show Z-stacks of fibers in Fig. 6. A single central longitudinal optical section through the axon is shown in Fig. 6A, while the entire stack is shown in Fig. 6B. Cross-sections (boxes in Fig. 6B) are shown in Fig. 6C, D, and E, demonstrating that the axons are indeed labeled and separated from the labeled Schwann cells by unlabeled compact myelin. A significant fraction of BrU was detected on the surface of the fiber, suggesting that the bands of Cajal (spirally shaped outer Schwann cell cytoplasm) contain newly-synthesized RNA (arrows).

**Figure 7:**
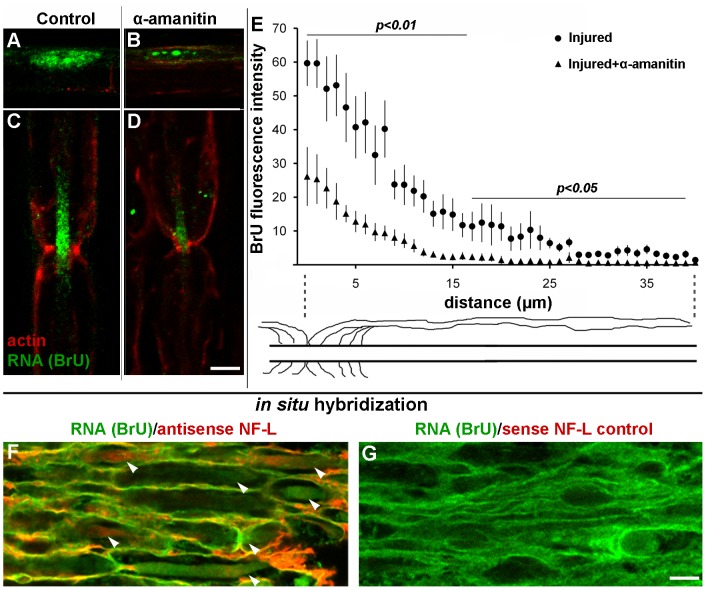
Most of the newly-synthesized RNA is produced by RNA Polymerase II. **A–E**, injured control nerves without α-amanitin (**A** **and** **C)**, and injured nerves treated with α-amanitin during the BrU labeling period **(B and D)** were stained for BrU (green) and F-actin with phalloidin (red). **A and** **B**, Schwann cell nuclei; **C** and **D**, nodes of Ranvier. Bar = 10 µm. **E**, BrU-RNA fluorescence intensities plotted as a function of distance from the node of Ranvier for controls without α-amanitin (circles) and nerves treated with 10 µg/ml α-amanitin (triangles). Statistical significance at each distance was determined by Student's t-test. Error bars represent standard errors. **F**, Neurofilament L (NF-L) mRNA is found in both Schwann cells and axons by *in situ* hybridization (red) and BrU-RNA (green). Arrows are pointing to axons. **G**, negative control NF-L sense probe. Bar = 5 µm.

To better classify the nature of the transferred axonal RNA, we performed the BrU labeling in the presence of 10 µg/ml alpha-amanitin, which inhibits RNA Polymerase II [26]. The labeling of Schwann cell nuclei (Fig. 7A and B) was reduced. Moreover, in treated fibers nucleoli labeled much more intensely than the rest of the nucleus, consistent with inhibition of transcription by RNA polymerase II, but not rRNA transcription. Inhibition with alpha-amanitin reduced the axonal BrU signal significantly (Fig. 7C and D). Quantitatively, mean measurements with standard error were 55.1±5.1 and 24.0±5.8 for control and alpha-amanitin respectively; the difference was significant at *p = *0.0007 by Student’s t-test. Pooled data are graphed in Fig. 7E, showing that a statistically-significant reduction in BrU intensity occurred throughout the gradient from nodes of Ranvier. These results are consistent with RNA polymerase II as the origin of a significant fraction of transferred RNA.

To assay for steady-state mRNA encoding a known axonal marker, we performed fluorescent *in situ* hybridization with an antisense probe to neurofilament-L (NF-L) mRNA. Our results showed that NF-L mRNA colocalizes with BrU-RNA in both Schwann cells and axons (Fig. 7F and G). While this experiment does not demonstrate cell-to-cell transfer, it is highly suggestive of transfer since NF-L protein was not detected in Schwann cells (data not shown).

### RNA Transfer is F-actin Dependent

**Figure 8:**
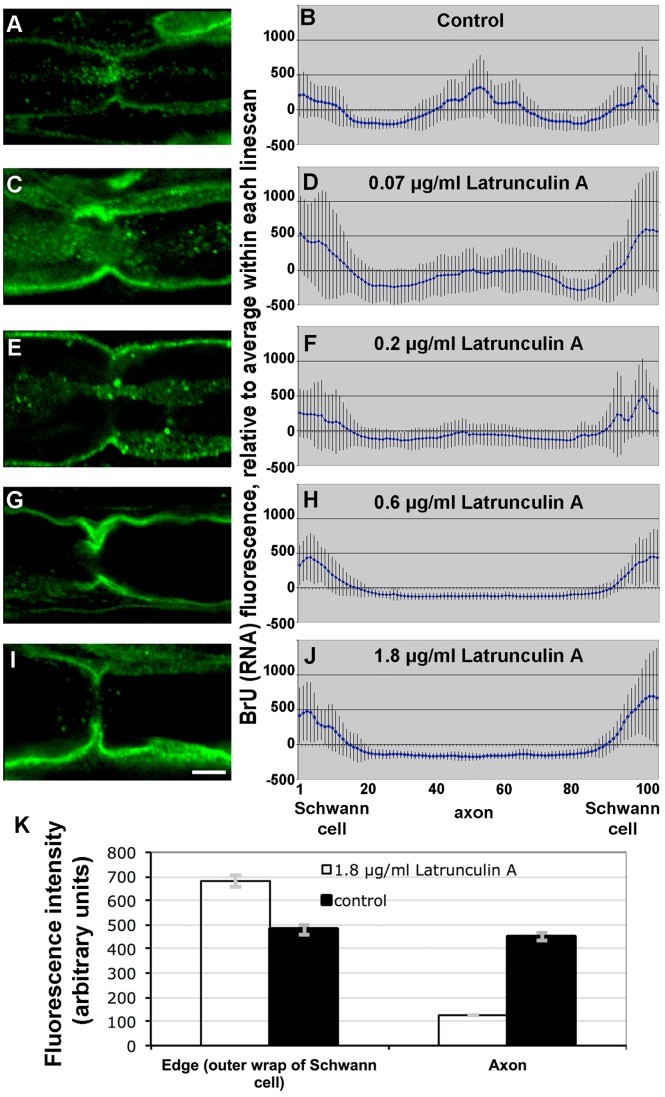
Actin depolymerization in injured sciatic nerves prevents transfer of RNA into axons. **A, C, E, G, I**, representative confocal images of BrU labeling at nodes of Ranvier. Bar = 10 µm. **B, D, F, H, J**, quantification of BrU fluorescence from 10 or more line scans across perinodal regions normalized to the mean of each linescan. Error bars represent standard errors. **A and B**, control BrU labeling without Latrunculin A; **C and D**, 0.07 µg/ml Latrunculin A during BrU labeling; **E and F**, 0.2 µg/ml; **G and H**, 0.6 µg/ml; **I and J**, 1.8 µg/ml. **K**, absolute BrU fluorescence intensities for the 8 bins at each edge combined (n = 304), representing RNA in the outer Schwann cell wrap, and the 20 bins in the center of each linescan (n = 380), representing RNA in the axon, for the control untreated and highest latrunculin A concentration (1.8 µg/ml) nerves. Error bars represent standard errors.

The high concentrations of newly-synthesized RNA in actin-rich regions suggested the involvement of actin in cell-to-cell transfer of RNA. To test this hypothesis, we depolymerized F-actin with 0.07–1.8 µg/ml latrunculin A. A representative labeled fiber at each concentration is shown in the left column of Fig. 8. Quantitation of axonal BrU labeling for each latrunculin A concentration is graphed in the right column of Fig. 8. While the graphs in the right column show normalized fluorescence intensities, the absolute intensities for control and 1.8 µg/ml latrunculin A samples are plotted in Fig. 8K. Student’s t-test of control vs. experimental intensities in edges and axons were significant with p = 0.02 and *p*<0.0001 respectively. In other words, the relative decrease of BrU signal in the axon was complemented by an increase of signal in the Schwann cells, consistent with inhibition of transport from the latter to the former.

### RNA Transfer is Myosin-Va-dependent

**Figure 9:**
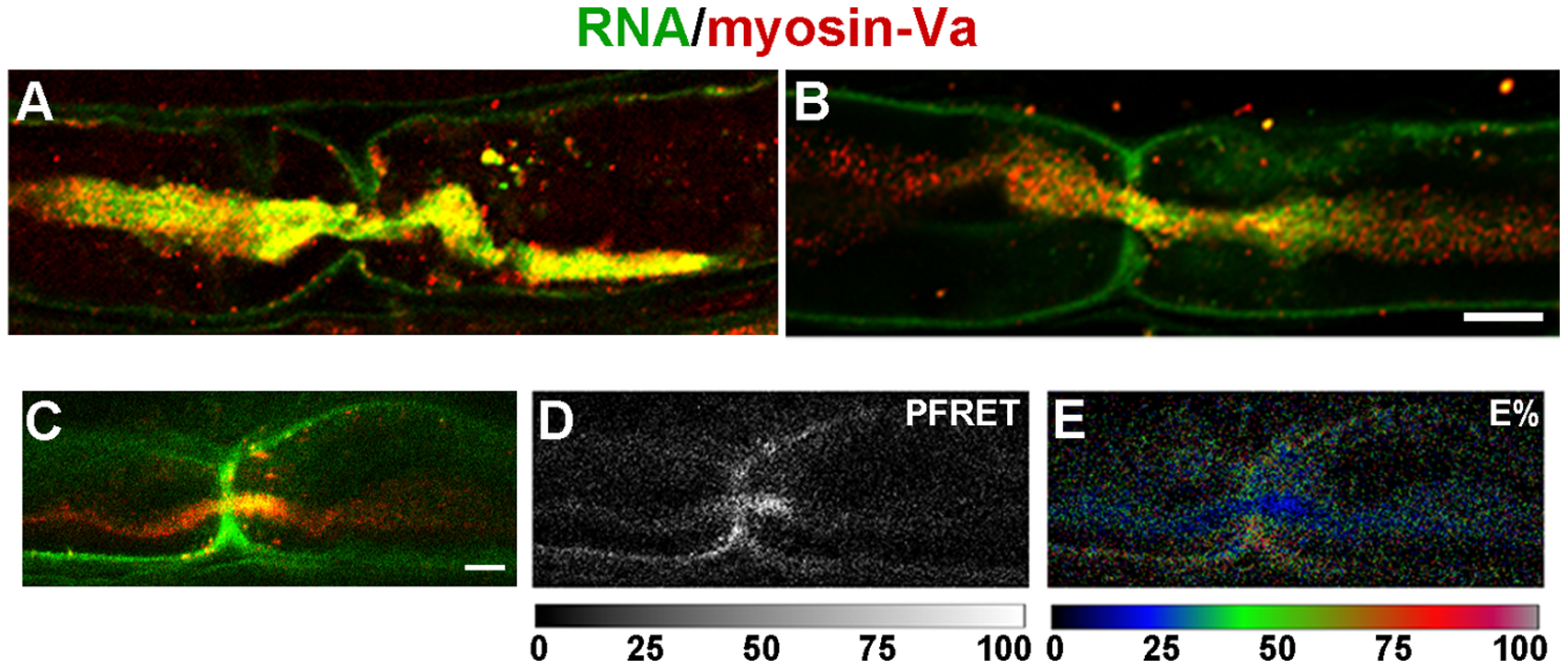
Colocalization of myosin-Va and newly-synthesized RNA in fibers of injured sciatic nerve axons. **A, B, and C**, confocal micrographs of nodes of Ranvier showing newly-synthesized RNA detected by BrU incorporation (green) and myosin-Va detected by immunofluorescence (red); **D**, processed FRET (PFRET) image for the node shown in panel C. **E**, FRET efficiency (E%) image for the node shown in panels C and D. Scales below panels D and E show lookup tables. Bars = 5 µm.

The requirement for actin in turn suggested a role for myosin motors, so we performed immunofluorescent detection of myosin-Va after transection. We observed significant colocalization of myosin-Va with newly-synthesized RNA (Fig. 9A–C). To estimate the distance between myosin-Va and newly-synthesized RNA, we performed quantitative fluorescence resonance energy transfer (FRET) between the secondary antibodies detecting the anti-myosin-Va and anti-BrU primary antibodies. The spectral bleedthrough-corrected processed FRET (PFRET) signal [24] was observed in axons and Schwann cell cytoplasm at the nodes of Ranvier (Fig. 8D). Specific FRET signals, as demonstrated by E%, an expression of distances between fluorophores of 1–10 nm, were enriched in axons near the nodes of Ranvier (Fig. 8E, Fig. S3 in File S1). Thus, our data are consistent with a close association of myosin-Va with BrU-RNA in axons.

**Figure 10:**
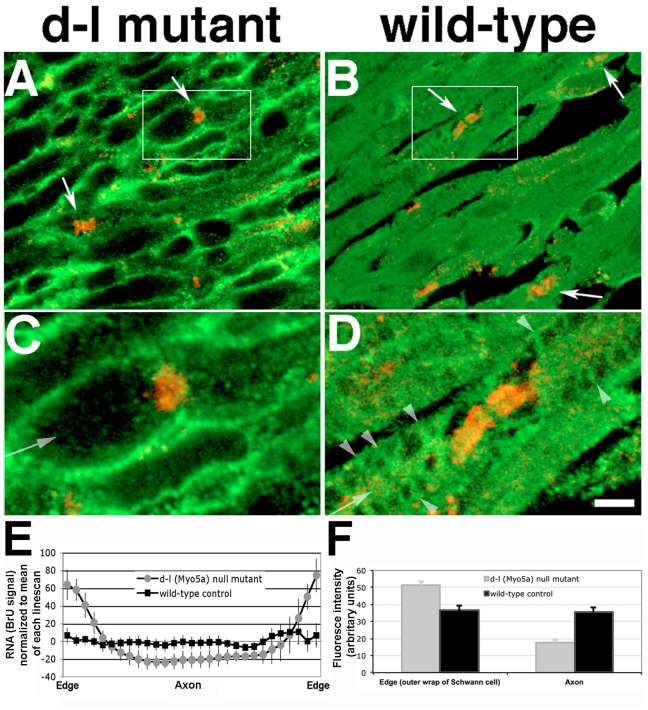
Myosin-Va function is required for transfer of RNA from Schwann cells to axons. Longitudinal 10-µm sections of transected sciatic nerves from null (d-l) *Myo5a* mutant mice have reduced axoplasmic levels of newly synthesized RNA. **A and C**, null mutant; **B and D**, wild-type control. RNA labeled by BrU is shown in green, the paranodal marker Caspr in red. Panels C and D show higher magnification views of boxed regions in panels A and B respectively. Arrows, nodes of Ranvier; arrowheads, bands of Cajal (compare to arrows in Fig. 6). Micrographs are single optical sections from Z-stacks imaged with a laser scanning confocal microscope. Bar = 5 µm. **E**, linescan quantitation of abundance of BrU-labeled RNA across fibers from d-l mutant and wild-type control mice. Edges are the outer wraps of Schwann cells; center approximates the location of the axon. Intensity measurements were normalized to the mean of each linescan. Bars represent standard deviations. **F**, Absolute BrU fluorescence intensities in edges (as shown in **E**, 4 bins at each end combined; n = 160) and centers (10 central bins combined; n = 200). Error bars represent standard errors.

As a genetic test for a requirement for myosin-Va function in cell-to-cell transfer of RNA, we modified the sciatic nerve transection and BrU labeling procedure developed for adult rats for 12–17-day-old mice, allowing us to perform the experiment on *dilute-lethal (Myo5a^d-l20J^/Myo5a^d-l20J^)* null mutant pups. These mice lack myosin-Va, which causes them to die at 19–21 days of age [27]. To compensate for the smaller diameter of mouse fibers, instead of teasing whole-mount preparations, the segments proximal to the transection were frozen and longitudinally sectioned. The results (Fig. 10) were striking: while wild-type littermate controls (Fig. 10B) had fibers and axons filled with BrU, as well as prominent labeling of bands of Cajal (Fig. 10D, arrowheads), axons of mutant mice had no detectable BrU labeling (Fig. 10A and C). Nodes of Ranvier (arrows) were identified by immunofluorescent detection of the paranodal marker Caspr [28]. To quantify the difference between mutant and wild-type fibers, we measured fluorescence intensities using 20-pixel wide linescans across 50 fibers chosen blindly from 5 mice of each genotype. There were two criteria: the first was greater width, to ensure a bias toward measuring diameters that included axons, and the second was doing linescans more than 100 µm from nodes of Ranvier, since the gradient observed in rat axons was not observed in mice. We normalized and binned the values, then graphed intensity by position along the lines (Fig. 10E), quantitatively demonstrating the lack of axonal labeling. While the values plotted in Fig. 10E were normalized to the mean of each linescan, differences in absolute intensities were apparent as well (Fig. 10F). Student’s t-tests of mutant vs. wild-type edges and axons both showed significant differences (*p*<0.0001). Thus, as with the latrunculin A inhibition experiment, the decrease in axonal signal is accompanied by an increase in Schwann cell signal, consistent with inhibition of transport, not synthesis.

Finally, we performed two controls to demonstrate that these observations was not an artifact of labeling an explanted nerve fragment. First, we performed the mice experiment described above using intraperitoneal injection of BrU with identical results (data not shown). Second, we performed the same experiment with intraperitoneal injection on 2-month-old wild-type mice (Fig. S4 in File S1), obtaining results similar to the results of the rat experiments, with gradients extending from nodes of Ranvier.

## Discussion

Evidence supporting the transfer of RNA and proteins from glia to axons, providing an additional source of macromolecules to the axon, has been previously reported but is not universally accepted. Pioneering work in the Mauthner axon of goldfish [9] suggested transfer of RNA. In mammals, autoradiography in rat sciatic nerve axons first suggested cell-to-cell transfer [10]. Biochemical assays following disruption of interactions between glia and squid giant axons [1], [11] further supported the transfer hypothesis in invertebrates. Ultrastructural studies of first internodal regions of motor axons [19] suggested cell-to-cell transfer of ribosomes, as they showed double-walled vesicles filled with what appear to be ribosomes at the glia-axon interface. This was supported by similar observations during the immunolocalization of ribosomes in sciatic nerve axons [20]. Van Minnen and coworkers used GFP tagging of a ribosomal protein expressed in a lentivirus to show that ribosomes assembled in Schwann cells were likely transferred to the axon [21], [22].

Here, by labeling newly-synthesized RNA at the site of axon injury in the complete absence of neuronal cell bodies, we have clearly shown that RNA is concentrated in the likely pathways for cell-to-cell transfer [6] at the nodes of Ranvier and Schmidt-Lanterman incisures (Figs. 2 and 3), that particles have been transferred into the axon, and that the cell-to-cell transfer is actin- and myosin-Va dependent. Our data complement the demonstration of tagged ribosomes in axons [21], [22], but there is an important difference: their experiments suggesting cell-to-cell ribosome transfer was done in axons distal to the injury site while our demonstration of cell-to-cell RNA transfer was documented directly at the regenerating end of axons proximal to the injury using a pulse-chase protocol in the complete absence of neuronal cell bodies. The labeling we observed is not an artifact of culturing the nerve fragments *in vitro* for three reasons: first, labeling *in situ* by leaving the injured nerve inside the rat thigh gave indistinguishable results (Fig. S2 in File S1); second, intraperitoneal injection of BrU in the mouse experiment gave the same results as explant culture of the nerve segment; and third, *in vivo* labeling with tritiated uridine gave similar results, but with far lower accuracy in location [10].

We do not yet know whether ribosomes and RNA are transferred separately or together, but our imaging of both (Fig. 3A) does not indicate complete coincidence. Moreover, our other experiments indicate that a large proportion of transferred RNA is likely to be mRNA, since most of the axonal RNA was absent in fibers treated with the RNA polymerase II inhibitor alpha-amanitin (Fig.7A–D). Both of these results indicate that our initial BrU experiments were not merely detecting ribosomal RNA. Moreover, we observed only very slight colocalization of BrU with mitochondrial markers, suggesting that very little or none of the newly-synthesized axonal RNA is of mitochondrial origin; nor is it taken up by mitochondria. More importantly, the BrU signal was lower in mitochondria. The BrU labeling was highly punctate and RNase-resistant, suggesting that ribonucleoprotein particles are transferred, similar to previous reports on cytoplasmic RNA transport within neuronal and non-neuronal cells [4], [29]–[31]. We also show that transcripts encoding a well-known neuronal marker protein, NF-L, are present at high levels in uninjured mouse sciatic nerve Schwann cells. While this experiment does not demonstrate cell-to-cell transfer, it is consistent with our model.

Our data begin to delineate the mechanism of cell-to-cell transfer of RNA from Schwann cells to axons, as we have clearly demonstrated that myosin-Va function is required for transfer (Fig. 10). There is an interesting parallel between this requirement and the requirement for myosin-Va function in another neural crest-derived system: the cell-to-cell transfer of melanosomes from melanocytes into hair bulbs and keratinocytes. It is important to note that while the absence of myosin-Va drastically alters melanosome transfer [32], there is no evidence directly implicating myosin-Va in the cell-to-cell transfer itself; more likely, its role may be limited to retaining melanosomes in the periphery of the melanocyte. We propose a similar mechanism in this case, with myosin-Va helping to retain RNA in the regions of the Schwann cell cytoplasm from which the transferred RNA is taken or donated. Whether the Schwann cell, axon, or both play the active role of cell-to-cell transfer remains an entirely open question.

There are three primary differences between the mouse data and the rat data. The first is the lack of any gradient of BrU immunoreactivity spreading out from the nodes of Ranvier. This is likely caused by a higher metabolic rate in the very young mice relative to that of the adult rats; shortening of the BrU labeling period to as little as 20 min did not produce a gradient (data not shown). Consistent with this hypothesis, labeling the injured sciatic nerves of 2-month-old, wild-type mice yielded similar results to the rat experiments (Fig. S4 in File S1). The second is the difficulty in distinguishing axons in the wild-type fibers, again due to the young age of the mice. The third difference is the thickness and raggedness of the Schwann-cell labeling in the mutant mice, likely because myelination is in progress at this age.

While the data presented here are from injured nerves (with the exception of the comparison of BrU gradients in Injured to Uninjured (Fig. 2) and *in situ* hybridization data in Fig. 7), they are provocative when combined with previous observations in uninjured axons: depolymerization of F-actin by cytochalasin B inhibits axonal protein synthesis [33], and that myosin-Va and the mRNA encoding it are present in periaxoplasmic ribosomal plaques in uninjured axons [34]. This raises interesting questions: first, is myosin-Va function required for axonal protein synthesis from mRNAs that originate in the neuronal soma; and second, does cell-to-cell transfer of RNA occur developmentally? We are addressing both questions using transgenic and knock-in mice with tissue-specific expression of tagged mRNAs and proteins.

In summary, these data confirm and extend our understanding of the complex relationship between glia and the axons they ensheath. This relationship is crucial in understanding mechanisms underlying responses to injury and neurodegeneration, as well as in designing therapeutic strategies that exploit intercellular transport for both retrograde signaling to the cell body [35] and controlling regeneration. The close associations and complex topologies of Schwann cell and axonal plasma membranes make assessment of intercellular transfer mechanisms difficult; however, our data suggest essential roles for both F-actin and myosin-Va in this mechanism.

## Supporting Information

File S1 **Contains Figures S1, S2, S3, and S4 with legends.**
(DOCX)

## References

[1] Lasek RJ, Gainer H, Barker JL (1977) Cell-to-cell transfer of glial proteins to the squid giant axon. The glia-neuron protein transfer hypothesis. J Cell Biol 74: 501–523. 885913 10.1083/jcb.74.2.501 PMC2110074

[2] Alvarez J (2001) The autonomous axon: a model based on local synthesis of proteins. Biol Res 34: 103–109. 11715201 10.4067/s0716-97602001000200014

[3] Brittis PA, Lu Q, Flanagan JG (2002) Axonal protein synthesis provides a mechanism for localized regulation at an intermediate target. Cell 110: 223–235. 12150930 10.1016/s0092-8674(02)00813-9

[4] Sotelo-Silveira JR, Calliari A, Kun A, Koenig E, Sotelo JR (2006) RNA trafficking in axons. Traffic 7: 508–515. 16643274 10.1111/j.1600-0854.2006.00405.x

[5] Van Horck FP, Holt CE (2008) A cytoskeletal platform for local translation in axons. Sci Signal 1: pe11. 18314505 10.1126/stke.18pe11 PMC3682639

[6] Twiss JL, Fainzilber M (2009) Ribosomes in axons–scrounging from the neighbors? Trends Cell Biol 19: 236–243. 19359177 10.1016/j.tcb.2009.02.007

[7] Crispino M, Cefaliello C, Kaplan B, Giuditta A (2009) Protein synthesis in nerve terminals and the glia-neuron unit. Results Probl Cell Differ 48: 243–267. 19554280 10.1007/400_2009_9

[8] Edstrom JE, Eichner D, Edstrom A (1962) The ribonucleic acid of axons and myelin sheaths from Mauthner neurons. Biochim Biophys Acta 61: 178–184. 13889252 10.1016/0926-6550(62)90080-4

[9] Jakoubek B, Edstrom JE (1965) RNA changes in the Mauthner axon and myelin sheath after increased functional activity. J Neurochem 12: 845–849. 5843551 10.1111/j.1471-4159.1965.tb10269.x

[10] Benech C, Sotelo JR, Menendez J, Correa-Luna R (1982) Autoradiographic study of RNA and protein synthesis in sectioned peripheral nerves. Exp Neurol 76: 72–82. 6177544 10.1016/0014-4886(82)90102-9

[11] Cutillo V, Montagnese P, Gremo F, Casola L, Giuditta A (1983) Origin of axoplasmic RNA in the squid giant fiber. Neurochem Res 8: 1621–1634. 6200785 10.1007/BF00964163

[12] Eyman M, Cefaliello C, Ferrara E, De Stefano R, Lavina ZS, et al (2007) Local synthesis of axonal and presynaptic RNA in squid model systems. Eur J Neurosci 25: 341–350. 17284174 10.1111/j.1460-9568.2007.05304.x

[13] Sotelo JR, Benech CR, Kun A (1992) Local radiolabeling of the 68 kDa neurofilament protein in rat sciatic nerves. Neurosci Lett 144: 174–176. 1436698 10.1016/0304-3940(92)90743-q

[14] Roberson MD, Toews AD, Goodrum JF, Morell P (1992) Neurofilament and tubulin mRNA expression in Schwann cells. J Neurosci Res 33: 156–162. 1453479 10.1002/jnr.490330120

[15] Kelly BM, Gillespie CS, Sherman DL, Brophy PJ (1992) Schwann cells of the myelin-forming phenotype express neurofilament protein NF-M. J Cell Biol 118: 397–410. 1321159 10.1083/jcb.118.2.397 PMC2290038

[16] Fabrizi C, Kelly BM, Gillespie CS, Schlaepfer WW, Scherer SS, et al (1997) Transient expression of the neurofilament proteins NF-L and NF-M by Schwann cells is regulated by axonal contact. J Neurosci Res 50: 291–299. 9373038 10.1002/(SICI)1097-4547(19971015)50:2<291::AID-JNR17>3.0.CO;2-B

[17] Sotelo-Silveira JR, Calliari A, Kun A, Benech JC, Sanguinetti C, et al (2000) Neurofilament mRNAs are present and translated in the normal and severed sciatic nerve. J Neurosci Res 62: 65–74. 11002288 10.1002/1097-4547(20001001)62:1<65::AID-JNR7>3.0.CO;2-Z

[18] Pearce J, Lnenicka GA, Govind CK (2003) Regenerating crayfish motor axons assimilate glial cells and sprout in cultured explants. J Comp Neurol 464: 449–462. 12900916 10.1002/cne.10828

[19] Li YC, Li YN, Cheng CX, Sakamoto H, Kawate T, et al (2005) Subsurface cisterna-lined axonal invaginations and double-walled vesicles at the axonal-myelin sheath interface. Neuroscience research 53: 298–303. 16129504 10.1016/j.neures.2005.07.006

[20] Kun A, Otero L, Sotelo-Silveira JR, Sotelo JR (2007) Ribosomal distributions in axons of mammalian myelinated fibers. J Neurosci Res 85: 2087–2098. 17520748 10.1002/jnr.21340

[21] Court FA, Hendriks WT, Macgillavry HD, Alvarez J, van Minnen J, et al (2008) Schwann cell to axon transfer of ribosomes: toward a novel understanding of the role of glia in the nervous system. J Neurosci 28: 11024–11029. 18945910 10.1523/JNEUROSCI.2429-08.2008 PMC6671360

[22] Court FA, Midha R, Cisterna BA, Grochmal J, Shakhbazau A, et al (2011) Morphological evidence for a transport of ribosomes from Schwann cells to regenerating axons. Glia 59: 1529–1539. 21656857 10.1002/glia.21196

[23] Wallrabe H, Chen Y, Periasamy A, Barroso M (2006) Issues in confocal microscopy for quantitative FRET analysis. Microsc Res Tech 69: 196–206. 16538626 10.1002/jemt.20281

[24] Chen Y, Periasamy A (2006) Intensity range based quantitative FRET data analysis to localize protein molecules in live cell nuclei. Journal of Fluorescence 16: 95–104. 16397825 10.1007/s10895-005-0024-1

[25] Elangovan M, Wallrabe H, Chen Y, Day RN, Barroso M, et al (2003) Characterization of one- and two-photon excitation fluorescence resonance energy transfer microscopy. Methods 29: 58–73. 12543072 10.1016/s1046-2023(02)00283-9

[26] Bensaude O (2011) Inhibiting eukaryotic transcription: Which compound to choose? How to evaluate its activity? Transcription 2: 103–108. 21922053 10.4161/trns.2.3.16172 PMC3173647

[27] Searle AG (1952) A lethal allele of dilute in the house mouse. Heredity 6: 395–401.

[28] Einheber S, Zanazzi G, Ching W, Scherer S, Milner TA, et al (1997) The axonal membrane protein Caspr, a homologue of neurexin IV, is a component of the septate-like paranodal junctions that assemble during myelination. J Cell Biol 139: 1495–1506. 9396755 10.1083/jcb.139.6.1495 PMC2132621

[29] Sossin WS, DesGroseillers L (2006) Intracellular trafficking of RNA in neurons. Traffic 7: 1581–1589. 17054760 10.1111/j.1600-0854.2006.00500.x

[30] Bassell GJ, Oleynikov Y, Singer RH (1999) The travels of mRNAs through all cells large and small. Faseb J 13: 447–454. 10064611 10.1096/fasebj.13.3.447

[31] Kosik KS, Krichevsky AM (2005) The elegance of the microRNAs: a neuronal perspective. Neuron 47: 779–782. 16157272 10.1016/j.neuron.2005.08.019

[32] Silvers WK (1979) The coat colors of mice. New York: Springer-Verlag. 379 p.

[33] Sotelo-Silveira J, Crispino M, Puppo A, Sotelo JR, Koenig E (2008) Myelinated axons contain beta-actin mRNA and ZBP-1 in periaxoplasmic ribosomal plaques and depend on cyclic AMP and F-actin integrity for in vitro translation. J Neurochem 104: 545–547. 17961153 10.1111/j.1471-4159.2007.04999.x

[34] Sotelo-Silveira JR, Calliari A, Cárdenas M, Koenig E, Sotelo JR (2004) Myosin Va and kinesin II motor proteins are concentrated in ribosomal domains (periaxoplasmic ribosomal plaques) of myelinated axons. J Neurobiol 60: 187–196. 15266650 10.1002/neu.20015

[35] Rishal I, Fainzilber M (2010) Retrograde signaling in axonal regeneration. Exp Neurol 223: 5–10. 19699198 10.1016/j.expneurol.2009.08.010

