# Supplementary material for: Myosin-Va-Dependent Cell-To-Cell Transfer of RNA from Schwann Cells to Axons
Source: PLoS One. 2013 Apr 23;8(4):e61905. doi: 10.1371/journal.pone.0061905 (PMC3633983; doi:10.1371/journal.pone.0061905)
Supplement: File S1 — Contains Figures S1, S2, S3, and S4 with legends. (DOCX) [file pone.0061905.s001.docx]

**Supporting Information File S1, Sotelo et al.**

**
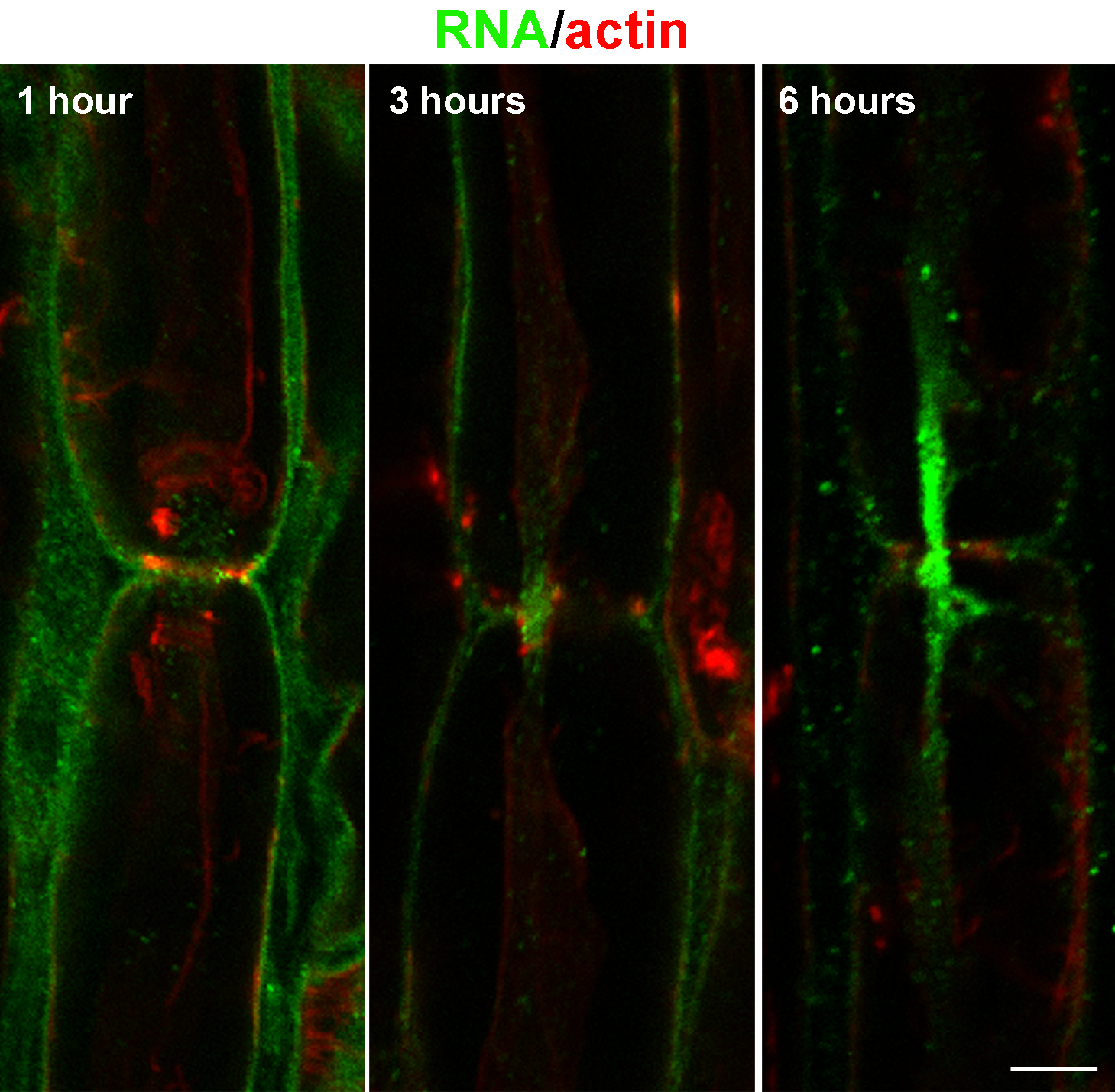
**

**Figure S1**

**Time course of bromouridine (BrU) labeling of newly-synthesized RNA.** Transected sciatic nerve fragments were obtained as shown in Fig. 2A-D except that they were incubated for 1 (left), 3 (middle), or 6 h (right) in BrU. Single confocal planes of single fibers at nodes of Ranvier show BrU incorporation (green) and F-actin (red).

**
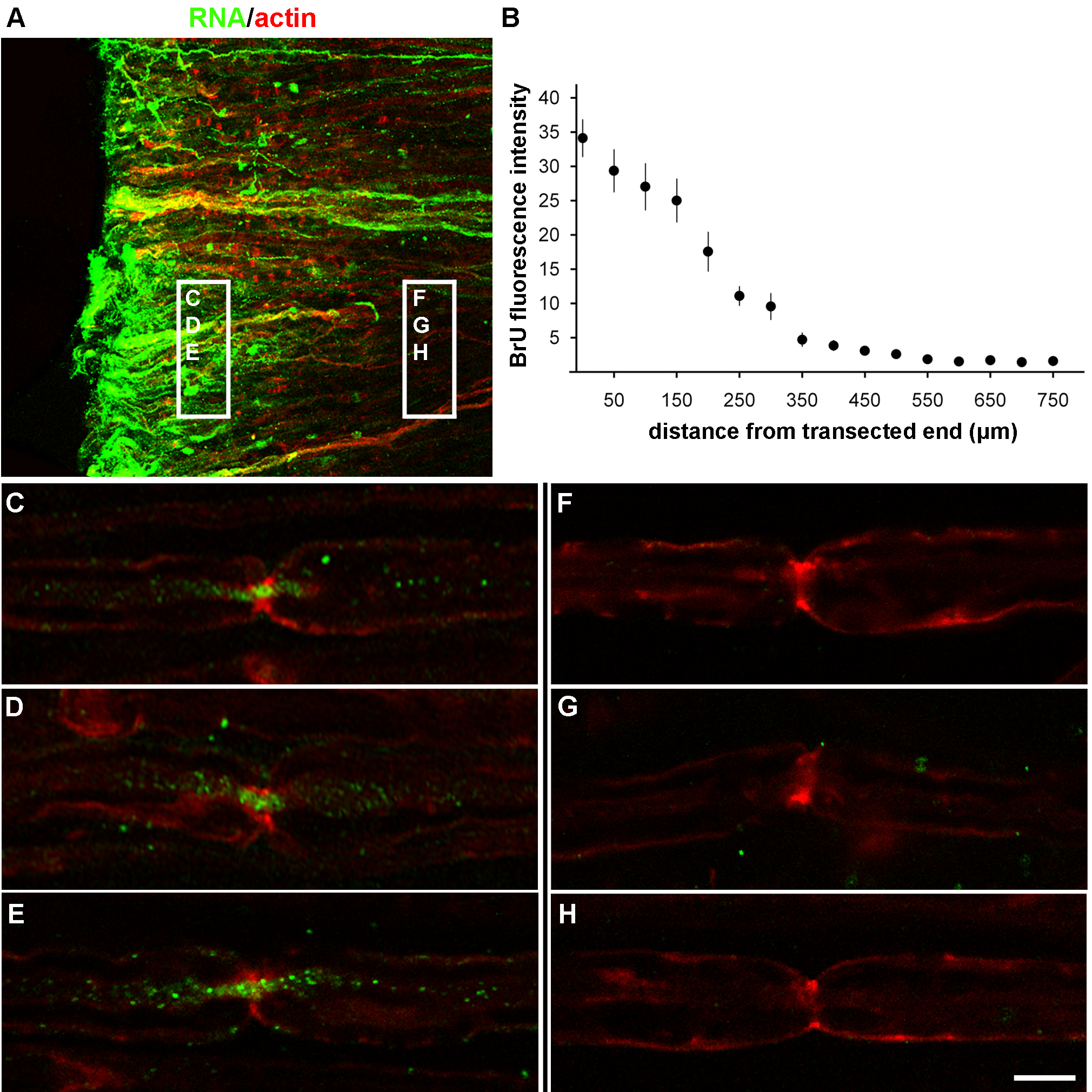
**

**Figure S2**

**Control *in vivo* labeling with BrU. A,** Low-magnification micrograph of transected end showing newly-synthesized RNA labeled by BrU incorporation (green) and actin labeled with phalloidin (red). Bar = 100 μm**.**  **B,** BrU-RNA signal plotted as a function of distance from the transection. Each point represents the mean of 10 nerve fragments with standard errors. **C-H**, series of higher-magnification images of fibers at nodes of Ranvier from the framed regions in panel. Bar = 10 μm**.**


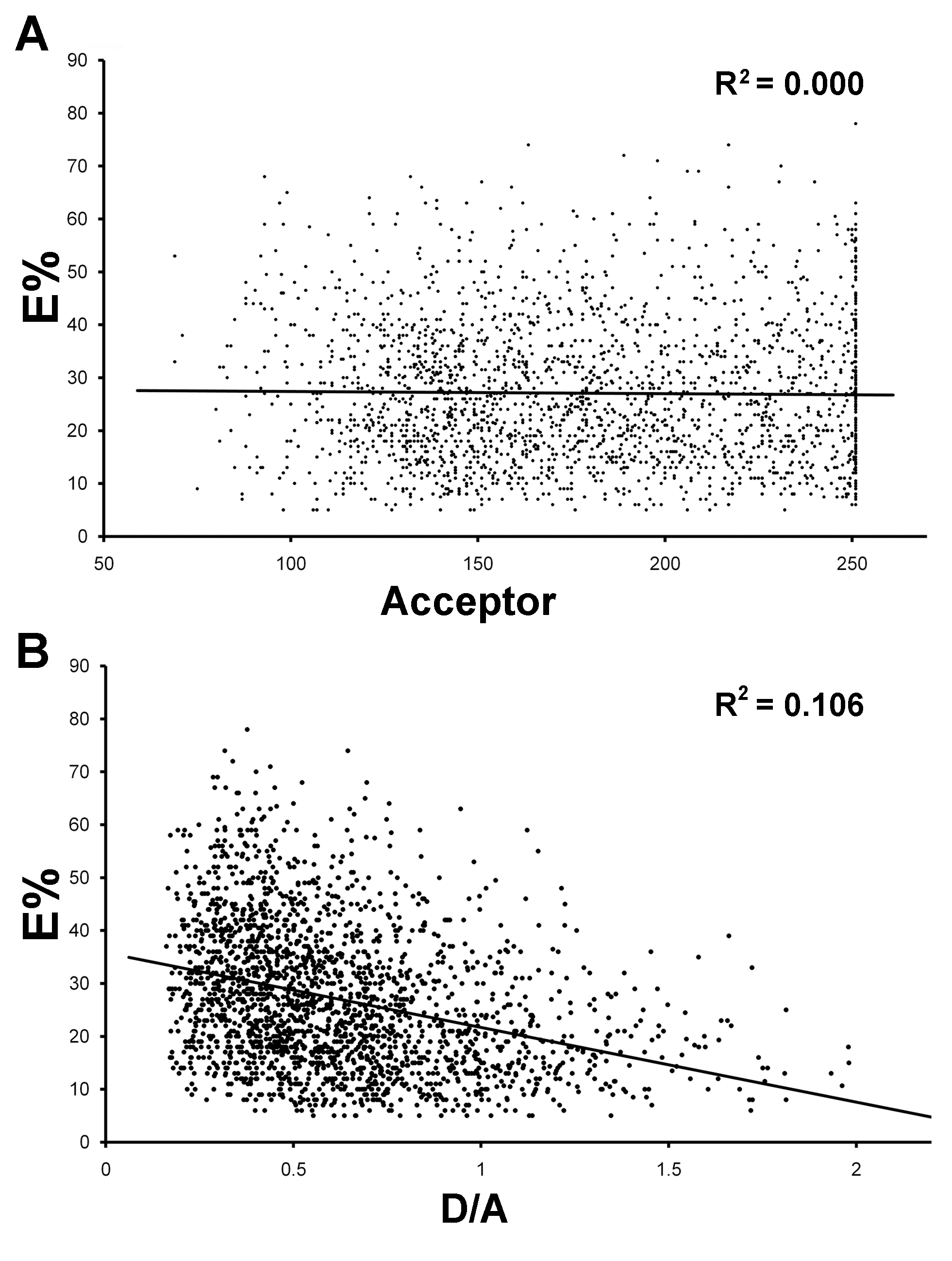


**Figure S3**

**Analysis of FRET parameters in axons at nodes of Ranvier.** A large database of regions of interest (ROIs -more than 2000), exclusively selected in axons at nodes of Ranvier, were assayed for E% and acceptor correlation (**A**) and E% and donor/acceptor ratio (D/A) correlation (**B**). **A.** R^2^ value = 0.000; statistically not significant, no correlation. **B.** R^2^ = 0.106; *p* < 0.000001. Trendlines and R^2^ were calculated in Excel software.

**
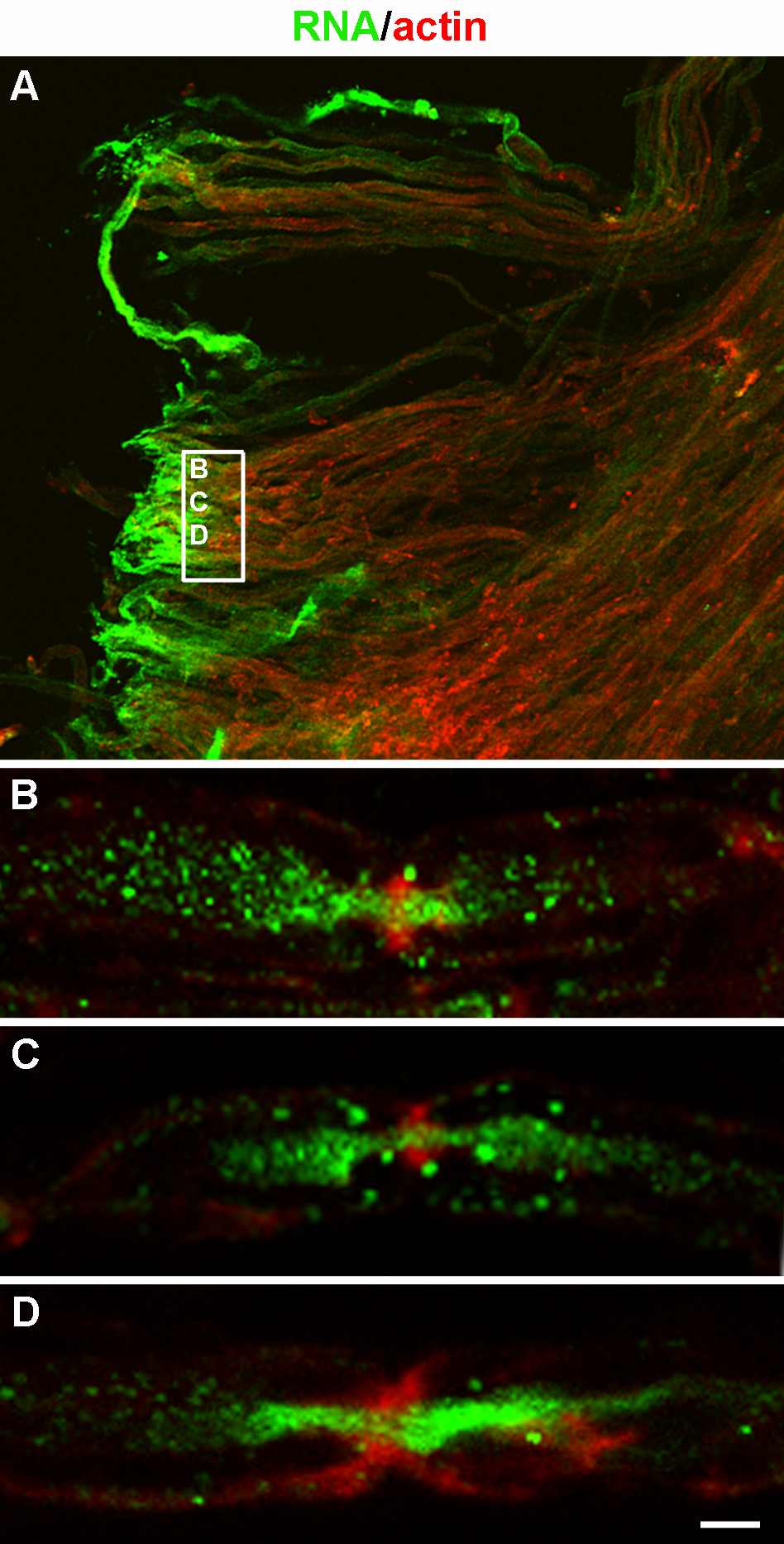
**

**Figure S4**

***In vivo* BrU labeling in adult mice. A,** Low-magnification micrograph of transected end showing newly-synthesized RNA labeled by BrU incorporation (green) and actin labeled with phalloidin (red). **B-D**, higher-magnification images of nodes of Ranvier from the framed region in panel A. Bar = 5 μm**.**
